# Oligomerization of the human immunodeficiency virus type 1 (HIV-1) Vpu protein – a genetic, biochemical and biophysical analysis

**DOI:** 10.1186/1743-422X-4-81

**Published:** 2007-08-29

**Authors:** Amjad Hussain, Suman R Das, Charu Tanwar, Shahid Jameel

**Affiliations:** 1 Virology Group, International Centre for Genetic Engineering and Biotechnology, New Delhi, India; 2 Laboratory of Viral Diseases, NIAID, NIH, Bethesda, MD, USA

## Abstract

**Background:**

The human immunodeficiency virus type 1(HIV-1) is a complex retrovirus and the causative agent of acquired immunodeficiency syndrome (AIDS). The HIV-1 Vpu protein is an oligomeric integral membrane protein essential for particle release, viral load and CD4 degradation. *In silico *models show Vpu to form pentamers with an ion channel activity.

**Results:**

Using Vpu proteins from a primary subtype C and the pNL4-3 subtype B isolates of HIV-1, we show oligomerization of the full-length protein as well as its transmembrane (TM) domain by genetic, biochemical and biophysical methods. We also provide direct evidence of the presence of Vpu pentamers in a stable equilibrium with its monomers *in vitro*. This was also true for the TM domain of Vpu. Confocal microscopy localized Vpu to the endoplasmic reticulum and Golgi regions of the cell, as well as to post-Golgi vesicles. In fluorescence resonance energy transfer (FRET) experiments in live cells we show that Vpu oligomerizes in what appears to be either the Golgi region or intracellular vesicles, but not in the ER.

**Conclusion:**

We provide here direct evidence that the TM domain, is critical for Vpu oligomerization and the most favourable channel assembly is a pentamer. The Vpu oligomerization appears to be either the Golgi region or intracellular vesicles, but not in the ER.

## Background

Primate lentiviruses, including HIV, encode a number of accessory proteins that perform essential functions during the viral life cycle [1]. One such protein is viral protein U (Vpu) that is encoded by HIV-1 but not HIV-2 or the simian immunodeficiency virus (SIV) [2]. Certain HIV-2 isolates have been shown to possess a partial Vpu-like activity in their envelope glycoprotein [3,4]. Whether Vpu is a viral pathogenesis factor remains to be established, but compared to HIV-1, closely related retroviruses such as HIV-2 and SIV that lack expression of a fully functional Vpu protein also cause less severe disease outcomes.

The Vpu protein of HIV-1 is an 81-amino acid type I integral membrane protein in which a short extracellular N-terminus is followed by a transmembrane (TM) domain and a cytoplasmic domain, the latter with two prominent alpha helices [2,5]. The region between the two helices, is highly conserved and contains two serine residues (S52 and S56) that are phosphorylated by cellular casein kinase II [6]. Two primary functions have been attributed to Vpu during the HIV-1 replication cycle. These include CD4 downmodulation and enhancement of viral particle release [7].

Like all retroviruses, HIV-1 also interferes with the expression of its cellular receptor and uses redundant mechanisms to achieve this [8]. The Vpu protein binds CD4 in the endoplasmic reticulum (ER) [9], and through its phosphoserine residues binds the beta transducin-repeat containing protein (βTrCP) in the cytoplasm [10]. The βTrCP recruits other proteins such as Skp1, Cul-1 and the Cdc34 E2 ubiquitin ligase [11]. This results in ubiquitination of CD4, its dislocation from the ER and degradation by the proteosome [12,13]. The stable association of Vpu with βTrCP also affects the latter's cellular functions, one of which is to direct the proteosomal degradation of inhibitor of kappa B (IκB) [14]. This results in inhibition of NFκB activity and the NFκB-dependent expression of anti-apoptotic genes of the Bcl-2 family [15]. Vpu also mediates the efficient release of viral particles from HIV-1-infected cells [16]. Though distinct from its CD4 degradation function, it is not clear whether Vpu enhances virus release through modification of the cellular environment or specific interactions with cellular or viral factors. The formation of conducting ion channels by Vpu [17] and its interaction with a novel tetratricopeptide repeat containing protein [18] favour both possibilities. The TM domain of Vpu has been shown to be important for enhancement of virus release [19,20] and pathogenicity [21]. This domain is also critical for its ion channel activity [13,17].

An earlier study has used chemical cross-linking to show that Vpu can form oligomers [22]. *In silico *modeling studies have predicted the same for the TM domain of Vpu [23]. The oligomeric nature of Vpu is also likely to affect its interaction with cellular proteins and therefore its role in HIV-1 pathogenesis. To better characterize the oligomerization of Vpu and the domains for this, we have used genetic, biochemical and biophysical methods, and two divergent Vpu proteins, one from a subtype C primary isolate and the other from a laboratory-adapted subtype B isolate of HIV-1. Our results show that Vpu or its TM domain form pentamers in solution. Using confocal microscopy and fluorescence resonance energy transfer experiments we further show that Vpu does not oligomerize in the ER, but does so in the Golgi region or in post-golgi vesicles.

## Results

### Cloning and expression of a functional Vpu

**Figure 1:**
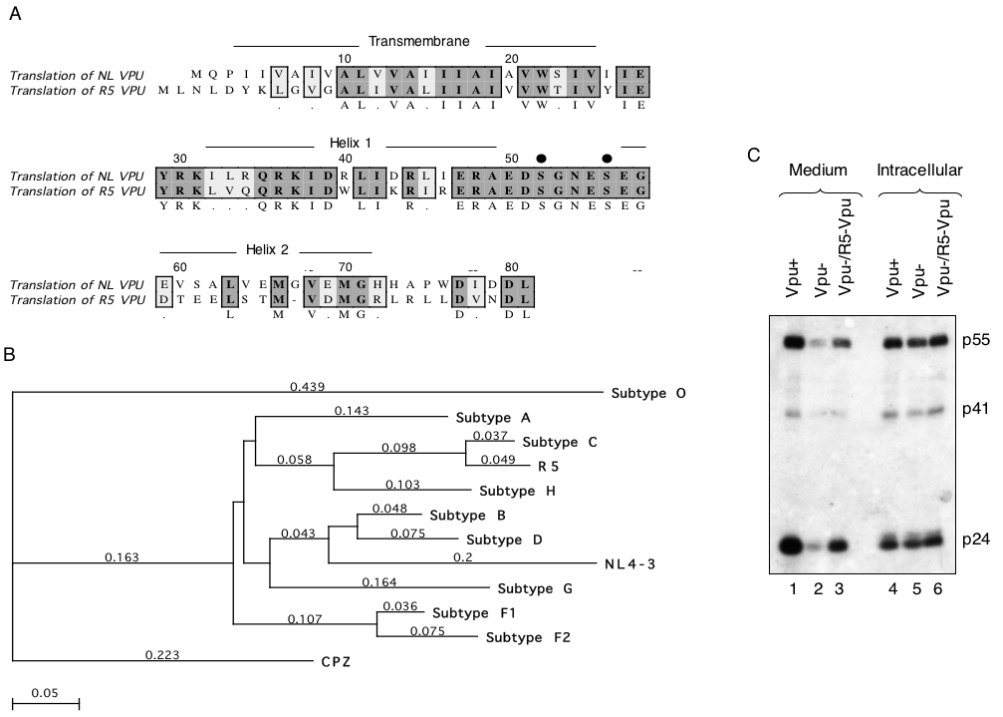
**Cloning and expression of functional Vpu**. (A) An alignment of the NL-Vpu (subtype B) and R5-Vpu (subtype C) protein sequences is shown. Boxes show sequence conservation; dark shading indicates sequence identity and light shading indicates conservative changes. The transmembrane domain, cytoplasmic helices and phosphoserine residues (black circles) are indicated. Residues are numbered according to the NL-Vpu sequence. (B) Phylogenetic comparison of the NL and R5 Vpu protein sequences to the consensus Vpu sequences for different HIV-1 subtypes available in the Los Alamos HIV sequence database http://www.hiv.lanl.gov/content/hiv-db/ALIGN_CURRENT/ALIGN-INDEX.html. The tree was drawn using the ClustalW (V1.4) algorithm in MacVector v7.2.2 (Accelrys). A Blosum similarity matrix was used with the following settings: Open Gap Penalty = 10; Extend Gap Penalty = 0.1; Delay Divergent = 40%; Gap Distance = 8. Numbers indicate the relative distance. (C) HeLa cells were transfected with wild type (Vpu+) or vpu-deficient (Vpu-) HIV-1 proviral DNA, the latter with empty vector (lanes 2 and 5) or an R5-Vpu expression vector (lanes 3 and 6), as described in Methods. Virions released in the culture medium (lanes 1–3) and intracellular virions (lanes 4–6) were estimated by western blotting with anti-p24 antibodies. The bands corresponding to the HIV-1 capsid precursors p55 and p41, and the mature p24 are indicated.

We used PCR to clone two *vpu *genes, one from the HIV-1 Subtype B lab-adapted isolate NL4-3 and the other from a HIV-1 Subtype C primary isolate from India (called R5). The translated amino acid sequences (Fig. 1A) showed the predicted TM domain, the cytoplasmic helices and the conserved serine residues of the Vpu protein. Multiple clones of R5 Vpu showed it to be 82 amino acids in length with two additional amino acids at the N-terminus and a deletion at residue 67 compared to NL Vpu. On comparing these sequences to those in the Los Alamos HIV database, Subtype C Vpu proteins were found to contain 2–5 extra amino acids at their N-terminus (Fig. 1A). Phylogenetic analysis of aligned sequences showed the R5 Vpu protein to be closest to the consensus sequence subtype C Vpu proteins in the database (Fig. 1B). Earlier, based on envelope heteroduplex mobility assay and the 3.5 kb *vpr-env *fragment sequence, we had determined the R5 primary isolate from India to belong to HIV-1 subtype C (SRD; unpublished). To ensure that the cloned *vpu *gene expressed a functional protein, a transfection-based HIV replication assay was set up. HeLa cells were transfected with wild type or *vpu*-deficient proviral DNA, the latter in the absence or presence of an *R5-vpu *expression vector. The virions released in the culture medium and those present within the cells were quantitated by western blotting with anti-Gag antibodies. As shown in Fig. 1C, while *vpu*-deficient proviral DNA produced as much virions as wild type proviral DNA (lanes 4 and 5), the release of virions into the culture medium was compromised in the absence of *vpu *(lanes 1 and 2). However, this phenotype was rescued following cotransfection of the *vpu*-deficient proviral DNA with the *R5-vpu *expression vector (lane 3). The effects were clearly visible at the level pf p55/p41 precursors as well as the p24 capsid protein. Thus, the R5 Vpu protein was functional in promoting virus release from cells.

### Homotypic and heterotypic interactions of full-length and truncated Vpu proteins

**Figure 2:**
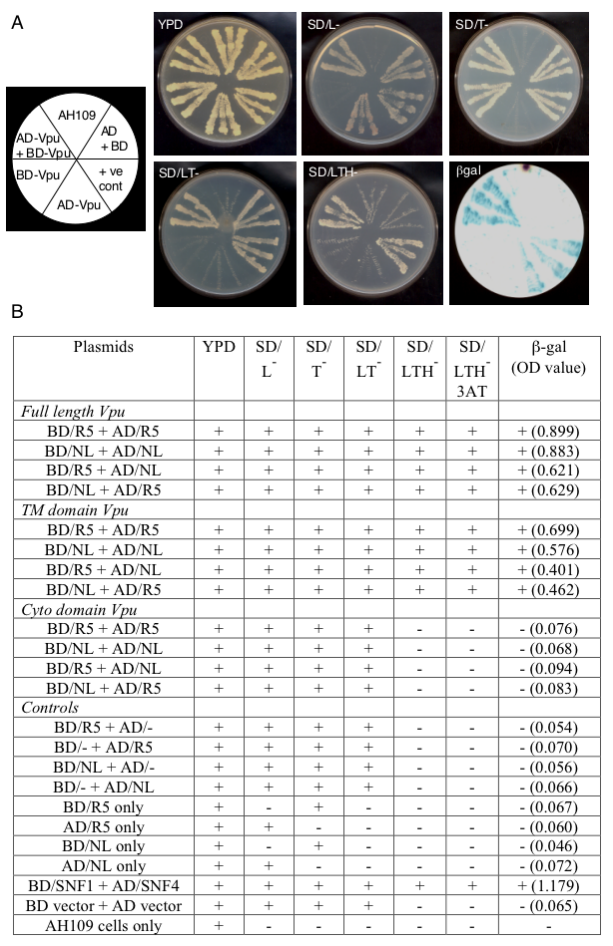
**Yeast two-hybrid analysis**. (A) Representative plates showing homotypic interactions of the R5-Vpu protein. The first panel shows the template for the remaining panels that in turn show transformants streaked in each section of the indicated plates. Growth is seen as light streaks on a dark background, except in the β-galactosidase filter assay where signal is seen as dark streaks on a light background. (B) Complete results for the entire screen using NL-Vpu and R5-Vpu full-length, transmembrane domain and cytoplasmic domain fusions to the Gal4 protein DNA-binding domain (BD) or activation domain (AD). Growth (+) or no growth (-) of transformants on various media is shown. LTH-3AT represents growth on SDLeu^- ^Trp^-^His^- ^plates containing 20 mM 3-amino-1,2,3-triazole. The β-galactosidase filter assay results are indicated as + or - and the liquid β-galactosidase assay values are shown in parentheses as an average of two independent measurements. Various negative and positive controls are also shown.

To test for the interaction between Vpu monomers, the full-length and truncated *vpu *genes were subcloned into the yeast two-hybrid expression vectors as fusions to the GAL4 DNA-binding domain (BD) and activation domain (AD). The expression of the full-length or truncated Vpu fusion proteins from these constructs was verified by T7 RNA polymerase mediated coupled *in vitro *transcription-translation (data not shown). The yeast two-hybrid assays were performed in *S. cerevisiae *AH109 cells as described in Materials and Methods. A representative set of plates is shown in Fig. 2A. All transformants grew on nonselective yeast extract-peptone-dextrose (YPD) plates (panel 2). Single transformants and all cotransformants containing AD-Vpu grew on SD/L^- ^plates (panel 3); similarly, those containing BD-Vpu grew on SD/T^- ^plates (panel 4) and cotransformants grew on SD/LT^- ^plates (panel 5). Cotransformants that contain interacting protein pairs fused to AD and BD can transactivate the *HIS3 *gene resulting in growth on SD/LTH^- ^plates. The growth of AD-Vpu/BD-Vpu cotransformants on a SD/LTH- plate (panel 6) showed homodimerization of the Vpu protein. Colonies were transferred to a nitrocellulose filter and a β-galactosidase filter assay was carried out. The presence of β-galactosidase activity only in the positive control and AD-Vpu/BD-Vpu cotransformants (panel 7) further confirmed the Vpu-Vpu interaction.

A more extensive screen, whose results are summarized in Fig. 2B, was carried out as above. Both NL Vpu and R5 Vpu showed homotypic interactions. Further, the NL Vpu and R5 Vpu proteins interacted with each other. All these interactions were found to depend upon the TM domains, but not on the cytoplasmic domains of Vpu. The transformants were grown in the presence of 20 mM 3-aminotriazole (3AT) to further confirm the specificity and strength of the interactions. All cotransformants that grew on SD/LTH^- ^plates also grew on SD/LTH^- ^3AT plates. Compared to the positive control (BD/SNF1+AD/SNF4), the semi-quantitative liquid β-galactosidase assay showed reasonably strong interactions between the full-length Vpu proteins and slightly weaker interactions between their TM domains. The values were higher for homologous interactions (NL vs NL and R5 vs R5) as opposed to heterologous interactions (NL vs R5). A variety of negative controls showed no interaction and only background β-galactosidase activity.

### Gel electrophoresis and in vitro binding assays

**Figure 3:**
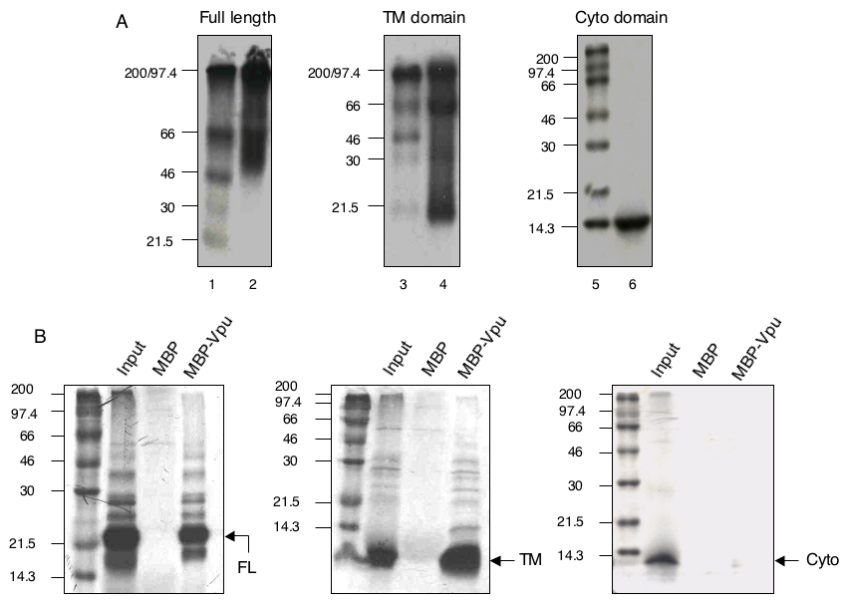
**Vpu oligomerization based on gel electrophoretic and pull-down assays**. The full-length R5-Vpu protein and its transmembrane and cytoplasmic domains were synthesized and labeled with ^35^S-methionine in a coupled *in vitro *transcription-translation system. (A) The proteins were analyzed on native polyacrylamide gels without heating or DTT treatment. Lanes 1,3 and 5, markers; lanes 2,4 and 6, Vpu full-length, TM domain and cytoplasmic domain, respectively. The molecular sizes (in kilodaltons) are indicated. (B) For pull-down assays, the ^35^S-labeled R5-Vpu proteins were synthesized *in vitro *and bound to amylose beads saturated with either the maltose binding protein (MBP)/R5-Vpu fusion protein or MBP alone as a control. The beads were washed, resuspended in loading dye buffer, boiled and the supernatants subjected to SDS-PAGE. The gels were dried and autoradiographed. Gels show the full-length (FL) R5-Vpu protein, or its transmembrane (TM) or cytoplasmic (cyto) domains, retained on the beads. Arrows indicate the full-length or truncated Vpu proteins.

We also tested the oligomerization of full-length Vpu (NL/R5) as well as its TM and cytoplasmic domains by polyacrylamide gel electrophoresis. The Vpu proteins were synthesized from the pGBK-Vpu and pGAD-Vpu plasmid templates using an *in vitro*-coupled transcription-translation assay (TNT; Promega, Madison, USA), in the presence of ^35^S-methionine. When these proteins were analyzed on native polyacrylamide gels (Fig. 3A), oligomeric species were prominently observed for full-length Vpu and its TM domain, but not for the cytoplasmic domain. A maltose binding protein (MBP)-Vpu fusion protein was expressed in *E. coli *and bound to amylose resin (New England Biolabs, Beverly, USA). The ^35^S-labeled *in vitro *synthesized Vpu proteins were then passed through these beads. Both full-length Vpu and its TM domain were retained on amylose beads saturated with MBP-Vpu, but not with the MBP control (Fig. 3B). The cytoplasmic domain of Vpu did not bind to MBP-Vpu in this assay. These results further support Vpu oligomerization through its TM but not the cytoplasmic domain.

### Vpu forms a pentamer in vitro

**Figure 4:**
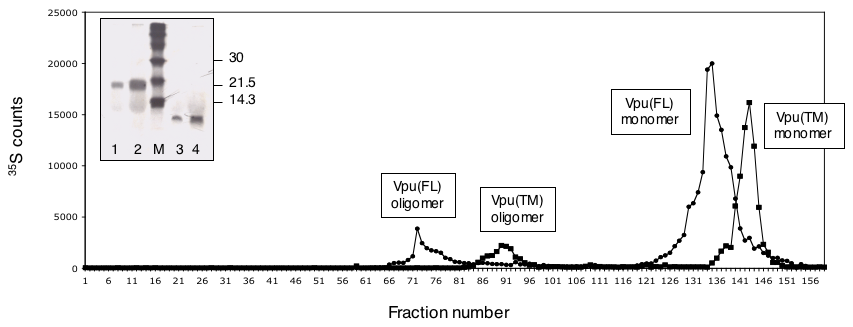
**Gel permeation analysis of Vpu oligomers**. *In vitro *translated and ^35^S-labeled Vpu proteins were separated by gel permeation chromatography as described in Methods. The eluted ^35^S counts in each fraction are indicated for the full-length (black circles) or TM domain (black squares) Vpu proteins. The positions of Vpu monomers and oligomers are indicated. The inset shows SDS-PAGE analysis of the peak fractions. Lanes: 1, Vpu oligomer; 2, Vpu monomer; 3, TM domain oligomer; 4, TM domain monomer; lane M shows molecular size marker as indicated (in kilodaltons).

Gel permeation chromatography of ^35^S-labeled full-length Vpu protein or its TM domain was carried out to characterize their oligomeric states. The proteins synthesized by *in vitro *coupled transcription-translation reactions were separated on a pre-calibrated Sephacryl S200HR column. Two prominent peaks of radioactivity were eluted for full-length as well as the TM domain proteins (Fig. 4). The faster eluting peak corresponding to the oligomer typically contained about 10% of the radioactivity. The peak oligomer and monomer fractions were further analyzed by SDS-PAGE to confirm the presence of Vpu (Fig. 4 inset). Based on their elution profiles, the calculated molecular masses were as follows: full-length Vpu monomer 15.3 kDa, oligomer 77.6 kDa; TM domain monomer 10 kDa, oligomer 47.8 kDa. Thus, the *in vitro *oligomers most closely represented pentamers for the full-length and TM domain Vpu proteins. These results also showed that Vpu monomers and pentamers existed in a stable equilibrium *in vitro *in the absence of other cellular components.

### Subcellular localization and FRET analysis

**Figure 5:**
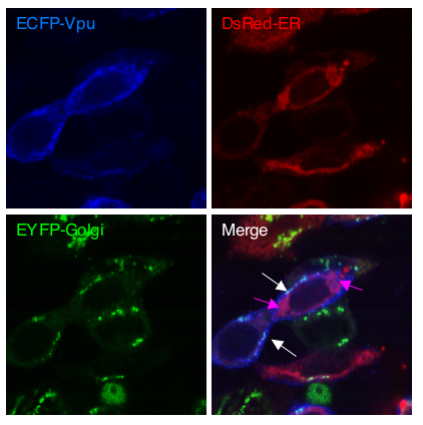
**Subcellular localization of Vpu**. U2-OS cells were cotransfected as described in Methods. The individual and merged images are shown. In the merged image, magenta and white arrows indicate Vpu/ER and Vpu/Golgi colocalizations, respectively.

It has been observed that Vpu localizes primarily to the cytoplasmic endomembrane structures in infected [16] as well as transfected cells [20]. We tested subcellular localization of the Vpu protein in transfected COS-1 and U2-OS cells. The cells were cotransfected with EGFP- or ECFP-vpu and either DsRed-ER, DsRed-mito or EYFP-Golgi expression vectors. The confocal images were sequentially acquired and merged for colocalization. In both cell types, the Vpu protein colocalized with the ER and Golgi markers (Fig. 5), but not with the mitochondrial marker (not shown). While a majority of the Vpu protein was found to be associated with the ER, significant amounts were also found to be associated with the Golgi.

**Figure 6:**
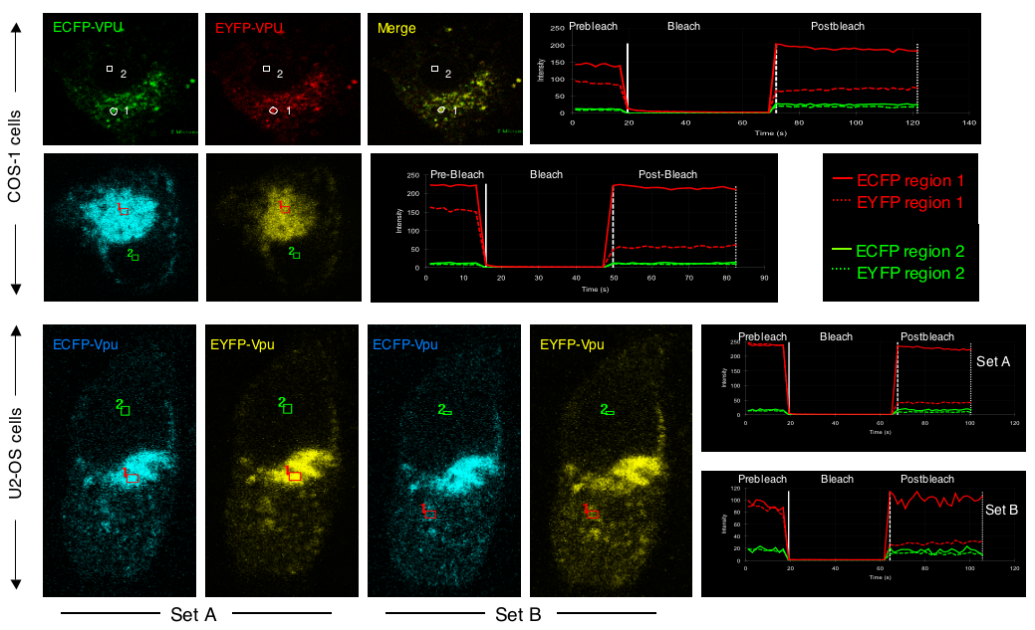
**FRET analysis of Vpu interactions**. COS-1 and U2-OS cells were cotransfected with Vpu-ECFP and Vpu-EYFP expression vectors and FRET was performed as described in Methods. Representative images for COS-1 and U2-OS cells are shown together with a kinetic profile of the FRET experiment. For U2-OS cells, sets A and B represent the same cell, with FRET carried out in two different regions of colocalization indicated by the box marked 1; the box marked 2 represents a control region of the cell. A total of 4 independent FRET experiments were carried out in 7–10 different cotransfected cells each time.

To detect intimate protein-protein interactions *in vivo*, we used fluorescence resonance energy transfer (FRET). This non-radiative energy transfer between donor and acceptor fluorophores is critically dependent upon the distance and dipole orientations of the two partners, and is taken as evidence of an interaction between them [24]. We cotransfected COS-1 or U2-OS cells with vectors expressing Vpu proteins fused to the cyan (ECFP) and yellow (EYFP) colored variants of the enhanced green fluorescent protein as the donor-acceptor FRET pair [25]. To measure FRET in cells, we followed an acceptor photobleach protocol wherein the mean fluorescence intensities from the donor (ECFP) and acceptor (EYFP) fluorophores were recorded before and after EYFP photobleaching. Two patterns of Vpu expression were observed in transfected COS-1 cells. In cells expressing low levels of the protein, a punctate and vesicular distribution was noted (Fig. 6; upper panels). However, cells expressing high levels of Vpu were found to accumulate this protein in intensely staining subcellular structures present on one side of the nucleus (Fig. 6; middle panels); these structures were also marked with the transfected DsRed-ER marker (not shown). Additionally, Vpu was also detected in more distal vesicular structures. In U2-OS cells, Vpu was similarly distributed in the ER, Golgi and punctate vesicular structures (Fig. 6; lower panels).

We carried out FRET measurements in live cells showing various patterns of Vpu subcellular distribution. Two different areas within the same cell, one showing colocalization and another where no colocalization was observed were subjected to FRET analysis. As expected (and seen in the merged image), the Vpu-ECFP and Vpu-EYFP proteins colocalized in transfected cells (Fig. 6; upper panels). On simultaneous scanning of the two fluorophores, there was an increase in cyan (donor) fluorescence following bleaching of the yellow (acceptor) fluorophore. Multiple FRET measurements were carried out in more than one region of the same cell with similar results (not shown). In COS-1 cells showing this pattern of Vpu distribution, the mean fluorescence intensities of ECFP-Vpu before and after EYFP-Vpu photobleaching were 111.95 ± 5.7 and 137.30 ± 6.2, respectively. This gave an average FRET efficiency of 18.5%. On the other hand, no FRET was observed in the ER region in COS-1 cells expressing high levels of tagged Vpu proteins (Fig. 6; middle panels). In U2-OS cells, the FRET analysis was carried out in two separate regions of the same transfected cells (Fig. 6; lower panels). No FRET was measured in the intensely staining ER region (Fig. 6, set A). However, FRET between ECFP-Vpu and EYFP-Vpu was observed reproducibly in regions of the cell that appeared to be either Golgi or unidentified vesicles (Fig. 6, set B). The mean fluorescence intensities of ECFP-Vpu before and after EYFP-Vpu photobleaching were 70.29 ± 4.26 and 95.04 ± 4.8, respectively. This gave an average FRET efficiency of 26%. For technical limitations in the imaging, it was not possible to also cotransfect these cells with subcellular markers to positively identify, in the same cell, those subcellular structures that support Vpu-Vpu FRET and those that do not. Overall, our FRET experiments provide strong *in vivo *evidence of Vpu-Vpu interaction in a live cell. Further, based on these results, Vpu appears to form oligomers in distinct subcellular locations, primarily in the Golgi and vesicular regions but not in the ER.

## Discussion

Lentiviruses encode a number of unique accessory proteins that are important for viral replication and pathogenesis, but are not encoded by other retroviruses. The versatility of HIV accessory proteins arise from their ability to function as adaptor molecules that connect various viral and cellular proteins to pre-existing cellular pathways, modulate these pathways and control processes important for viral replication. Thus, protein-protein interactions are an important aspect of the functioning of these proteins. The Vpu protein of HIV-1 is known to bind CD4 and βTRCP, the latter being a component of the E3 ubiquitin ligase complex. This association is instrumental in dislocation of CD4 from the ER, its ubiquitination and subsequent degradation by the proteosome [10]. The other function of Vpu is to promote the release of progeny viruses from infected cells. This appears to be dependent upon the ability of Vpu to form oligomeric complexes with an ion channel activity in cellular membranes [7].

The nature of the Vpu oligomer is important due to its functional significance in virion release. An earlier study used chemical cross-linking to demonstrate the oligomerization of Vpu [22]. Here we have used various genetic, biochemical and biophysical approaches to characterize the oligomerization of Vpu *in vitro *and *in vivo*. Molecular dynamic simulations and conductance studies have shown that the Vpu TM domain is sufficient for its ion channel activity [26] and the pattern of channel activity is characteristic of the self-assembly of conductive oligomers in the membrane [27]. This suggested that the TM domain of Vpu would also be required for its oligomerization. We provide here direct evidence that the hydrophobic N-terminal TM domain, and not the charged cytoplasmic domain, is critical for Vpu oligomerization. The two-hybrid and MBP pull-down analyses further showed that full-length Vpu proteins as well as their TM domains derived from two different HIV-1 subtypes interacted efficiently with each other. The isolated TM domains also showed stable interaction with the full-length Vpu protein. Molecular-dynamic simulations of ion channels formed by the Vpu TM domain predict the most favourable channel assembly to be a pentamer, but higher and lower oligomeric species were also predicted [23,28]. Recently, Becker et al [29] have used synthetic proteins containing a carrier template to which four or five peptides corresponding to the Vpu TM domain were attached, to demonstrate ion channel formation by oligomerization of the TM domain. In our analysis, multimeric species, including pentamers were evident on gel electrophoresis of the *in vitro *synthesized Vpu proteins and their TM domains. Further, molecular sizing of *in vitro *synthesized Vpu by gel permeation chromatography clearly showed it to assemble into a pentameric species. Thus, while complementing earlier studies [23,28,29] we provide direct evidence for pentamerization of the full-length Vpu protein as well as its TM domain. *In vitro *synthesized Vpu proteins, in the absence of other cellular components, demonstrated a stable equilibrium between monomers and pentamers. This demonstrates the inherent ability of Vpu to oligomerize and pentamers appear to be the thermodynamically most stable form of these oligomers.

Earlier studies have shown Vpu to be localized to the perinuclear region of the cell that includes the ER and Golgi [16,20]. Using CD4-Vpu fusion proteins and endoglycosidase H resistance, an earlier study has provided evidence for Vpu movement beyond the ER [30]. A similar fusion protein was also used to tease out the apoptotic pathway [15]. Recently, the imaging of a Vpu-EGFP fusion protein has also localized it to the ER, Golgi and plasma membrane [31]. So, there is evidence that Vpu has the ability to be transported to post-ER membranes. Several models have been proposed for a role of the Vpu channel in the budding of new virions [27]. It has been suggested that oligomerization of Vpu at the ER could form conducting channels leading to a collapse in the membrane potential across the ER cisternae and acceleration of membrane fusion and protein traffic in the exocytic pathway. Alternatively, at the ER/mitochondrial junctions, Vpu is proposed to collapse of the mitochondrial membrane potential and promote apoptosis. It is also possible that Vpu channels in the plasma membrane may attenuate the cell resting potential, promoting the fusion and release of new virions. We have used confocal microscopy and FRET analysis to test these models. Using transfected fluorescent protein-tagged Vpu fusion proteins and subcellular markers we show that Vpu localizes to the ER and Golgi regions but not to the mitochondria. In the absence of its mitochondrial localization, it would be difficult to support a direct effect of Vpu on the mitochondrial pore transition complex or transmembrane potential [32]. One possibility is the effect of Vpu channels on Ca^2+ ^release from its intracellular stores in the ER [33]. The FRET analysis in this study showed no oligomerization of Vpu associated with the ER, arguing against the role of ER directly or indirectly in this process.

Oligomerization of Vpu was observed by FRET in structures that were distal to the ER. While these structures could not be identified positively in the same cell due to the technical limitations, separate stainings in two different cell types (COS-1 and U2-OS) suggested that these structures might either be Golgi or vesicles associated with exocytic protein transport. The Vpu ion channel activity would be detrimental to ER function, inducing ER toxicity, stress and apoptosis of the infected cell. This would go against the plan of survival of productively infected cells followed by retroviruses and so nicely exemplified by HIV [34]. It would therefore make sense for Vpu oligomerization to occur downstream of the ER, enroute to the plasma membrane. Our FRET analysis in live cells supports this model. Since the cytoplasmic and not the transmembrane domain of Vpu is required for CD4 relocation from the ER and its subsequent degradation [20], this scheme would not affect the CD4 downmodulation function of Vpu. However, contrasting results have recently been presented wherein Vpu with a scrambled transmembrane domain was unable to downmodulate CD4 from the surface of transfected cells [21]. Whether this is due to the inability of mutant Vpu to oligomerize, or due to an altered protein structure or its arrangement in the membrane, remains to be seen.

## Conclusion

We have used genetic, biochemical and biophysical methods to complement earlier studies on Vpu oligomerization and the role of its N-terminal transmembrane domain in this oligomerization. While theoretical modeling studies [23,28] and synthetic peptides [29] had earlier predicted pentameric Vpu channels, we provide here direct evidence for the existence of a Vpu pentamer in stable equilibrium with its monomer. This was also true for the Vpu transmembrane domain. Finally, subcellular localization and FRET analysis argue against an earlier model of Vpu-mediated virion release based on channel formation in the ER. Besides channel formation and its effect on virion release, oligomerization would also influence the ability of Vpu to interact with host cell proteins towards regulating the intracellular environment for efficient viral replication, assembly and release. We are currently targeting this aspect of Vpu biology by screening for novel cellular partners.

## Methods

### Cloning and expression of vpu

The *vpu *gene was PCR amplified with Pfu polymerase (Stratagene, La Jolla, USA) using as template either the pNL4-3 plasmid DNA (NIH AIDS Research and Reference Reagent Program) or a 3.5 kb fragment encompassing the *vpr *to *env *region previously amplified and cloned from a primary isolate of HIV-1 subtype C (SRD, unpublished). The PCR primers used were as follows (with the restriction sites in italics): For the NL4-3 *vpu *gene, Vpu-NL-F, *GGATCC*ATGCAACCTATAATAGTA GCAATA and Vpu-NL-R, *GAATTC*ACTACAGATCATCAATATCCCAAG; for the R5 *vpu *gene, Vpu-R5-F, *GGATCC*ATGTTAAATTTAGATTATAAATTAGGAGTA GG and Vpu-R5-R, *GAATTC*ATTACAAATCATTAACATCCAAAAGCC. The amplified fragments designated as NL *vpu *and R5 *vpu *respectively, were cloned in plasmid pGEMT-Easy (Promega, Madison, USA) and sequenced in both directions. The gene fragments corresponding to the transmembrane (TM) domains were assembled from the following synthetic oligonucleotides: For the NL4-3 *vpu *TM region, NL-TM-F, CATGGAGATGCAACCTATAATAGTAGCAATAGTAGCATT AGTAGTAGCAATAATAATAGCAATAGCTGTGTGGTCCATAGTAATCATAGAATAGG and NL-TM-R, AATTCCTATTCTATGATTACTATGGACCACACAGCTATTGCTATTATTATTGCTACTACTAATGCTACTATTGCTACTATTATAGGTTGCATCTC; for the R5 vpu TM region, R5-TM-F, CATGGAGATGTTAAATTTAGATTATAAATTAGGAGTAGGAGCATTGATAGTAGCACTAATCATAGCAATAGTCGTGTGGACCATAGTATATATAGAATAGG and R5-TM-R, AATT CCTATTCTATATATACTATGGTCCACACGACTATTGCTATGATTAGTGCTA CTATCAATGCTCCTACTCCTAATTTATAATCTAAATTTAACATCTC. The cytoplasmic domains were PCR amplified using specific primers for NL *vpu *and R5 *vpu *as follows: for the NL *vpu *cytoplasmic region, NL-Cyto-F, *CCATGG*AGTATAGGAAAATATTAAGA and Vpu-NL-R (shown above); for the R5 *vpu *cytoplasmic region, R5-Cyto-F, *CCATGG*AGTATAGGAAATTGGTACAAC and Vpu-R5-R (shown above).

### Vpu functional assay

Eighteen to 24 hr prior to transfection, 0.3 × 10^6 ^HeLa cells were plated per 60 mm dish. These were cotransfected with 2 μg of either wild type or *vpu*-deficient HIV-1 proviral DNA and 0.5 μg of the expression vector pEGFP/R5-Vpu using Lipofectin (Clontech). As a control, the empty vector pEGFP-N1 was used. Twenty-four hr post-transfection, the released virions in the culture medium were pelleted through a 20% sucrose cushion for 2 hr at 100,000 × g in a Beckman SW41 rotor. The pelleted virions and harvested cells were lysed in Laemmli sample buffer. Proteins were separated by electrophoresis on SDS-10% polyacrylamide gels and western blotted with an anti-p24 antibody.

### Yeast two-hybrid assays

The GAL4-based two-hybrid system contained the DNA binding domain vector pGBKT7 and the activation domain vector pGADT7. The NL and R5 *vpu *genes were cloned into the pGBKT7 and pGADT7 vectors as *EcoRI-BamHI *fragments from the pGEMT-Easy clones. The nucleotide sequences corresponding to the TM and cytoplasmic domains of the Vpu proteins were similarly cloned in the two-hybrid vectors as *NcoI-BamHI *fragments, respectively. Expression of the relevant fusion proteins from each of the Vpu two-hybrid constructs was checked in a T7 polymerase based *in vitro *coupled transcription-translation system (Promega, Madison, USA) followed by immunoprecipitation with anti-Vpu antibodies. The yeast two-hybrid analysis was essentially carried out as described earlier [35,36]. Plasmids pGBKT7-vpu (BD/NL or BD/R5) and pGADT7-vpu (AD/NL or AD/R5) plasmids were cotransfected into *Saccharomyces cerevisiae *strain AH109 (*MAT***a ***trp1-901 his3 leu2-3, 112 ura3-52 ade2 gal4 gal80URA3*::*GAL-lacZ LYS2*::*GAL-HIS3*) containing the *HIS3 *and *lacZ *reporter genes under the control of GAL4-binding sites. The host strain containing plasmids pAS2-SNF1 and pACT2-SNF4 was used as a positive control [37]. Various negative controls that included single or dual transformants were also run in the same assay. The AH109 yeast cells were transformed using the lithium acetate procedure and plated on either complete YPD medium or synthetic dextrose (SD) in the absence of either leucine (SD/L-) or tryptophan (SD/T-), or both (SD/LT-). Protein interaction was tested by growth on SD plates without leucine, tryptophan and histidine (SD/LTH-) and the specificity of the interaction was tested as growth on SD/LTH- plates containing 20 mM 3-amino-1, 2, 3-triazole (SD/LTH-3AT). The β-galactosidase filter-lift assay was carried out as described earlier [35,36]. A semi-quantitative liquid β-galactosidase assay was carried out using the substrate chlorophenol red-β-D-galactopyranosidase as described elsewhere [38].

### In vitro expression and analysis

The *in vitro *expression of full-length Vpu or its TM or cytoplasmic domains was carried out using a coupled transcription-translation system (TNT; Promega, Madison, USA) as recommended by the supplier. The proteins were labeled with ^35^S-methionine in the same reaction and their authenticity was checked by immunoprecipitation with anti-Vpu antibodies. Five μl of the *in vitro *expression mix was analyzed by electrophoresis on 12% native polyacrylamide gels.

### Pull-down assays

The *vpu *gene was cloned as a *BamHI-EcoRI *fragment into the pMal-c2 vector (New England Biolabs, Beverly, USA) and the maltose-binding protein-Vpu fusion (MBP-Vpu) protein was expressed in BL21(DE3) cells. Following induction of a freshly diluted overnight culture with 1 mM IPTG for 4 hr at 37°C, the cells were harvested and resuspended in PBS containing 0.1% Triton-X100, 10 μg/ml lysozyme and 1 mM PMSF, and subjected to 5 cycles each of freeze-thaw and sonication. The sample was centrifuged at 12,000 rpm at 4°C in a microfuge (Biofuge 17RS, Heraeus). To the clarified lysate, amylose resin (New England Biolabs, Beverly, USA) was added and the MBP-Vpu protein was allowed to bind for 2 hr at 4°C. The resin was washed with 10 volumes of wash buffer containing 20 mM Tris-HCl, pH 7.4, 0.2 M NaCl, 10 mM β-mercaptoethanol, 1 mM EDTA to remove non-specifically bound proteins. To 50 μl of amylose beads saturated with either MBP or MBP-Vpu, 25 μl of ^35^S-labeled Vpu (prepared using a coupled *in vitro *transcription-translation system) diluted in 500 μl PBS was added and allowed to bind for 3 hr at 4°C with end-over mixing. The beads were then centrifuged down and washed five times with 500 μl each of PBS containing 0.1% Triton X-100, resuspended in SDS dye loading buffer and subjected to SDS-PAGE and fluorography.

### Gel permeation chromatography

The oligomeric forms of Vpu were also analyzed by gel permeation chromatography on Sephacryl S200HR (Pharmacia-Amersham). Following *in vitro *expression and ^35^S labeling, 50 μl of the reaction mixture was loaded on a 36 ml column pre-equilibrated in PBS. The column was run at a flow rate of 0.4 ml/min and 0.25 ml fractions were collected. The elution of Vpu was estimated by liquid scintillation counting and SDS-PAGE analysis of the peak radiolabeled fractions. For molecular size estimations, the column was calibrated with lysozyme and BSA under the same run conditions.

### Confocal microscopy and FRET assays

For confocal microscopy and FRET, the NL and R5 *vpu *genes were cloned as *EcoRI-BamHI *fragments in the Living Colors™ vectors pEGFP-N3, pEYFP-N1 and pECFP-N1 (Clontech). Prior to this, the genes were first PCR amplified, cloned in the pGEMT-Easy vector and sequenced. The following PCR primers (with restriction sites shown in italics) were used: for NL *vpu*, NL-X-F, *GAATTC*ATGCAACCTATAATAGTAGCAATA and NL-R-GFP, *GGATCC*GCG CAGATCATCAATATCC; for R5 *vpu*, R5-X-F, *GAATTC*ATGTTAAATTTAG ATTATAAATTAGGAGTAGG and R5-R-GFP, *GGATCC*TGCCAAATCATT AACATCCAAAA. For colocalization experiments, COS-1 and U2-OS cells were seeded at about 50% confluency on coverslips in 12-well plates, grown for 18 hr, and then cotransfected with Living Colors™ vectors expressing Vpu and any of the subcellular markers. At 24 to 48 hr post-transfection, the PBS-washed cells were fixed with 2% paraformaldehyde in PBS at room temperature for 10 min. These were then mounted using Antifade (Bio-Rad, Hercules, USA) and sealed. Confocal images were collected sequentially using a 60 planapo NA 1.4 objective on a Radiance 2100 laser scanning system (Bio-Rad, Hercules, USA) equipped with a Nikon Eclipse TE2000-U microscope. For FRET analysis, COS-1 and U2-OS cells were similarly transfected with ECFP-vpu and EYFP-vpu expression plasmids. The ECFP-Vpu (FRET donor) and EYFP-Vpu (FRET acceptor) images were acquired sequentially in live cells using the Blue diode 405 nm and the Argon ion 514 nm laser lines, respectively. Images of the ECFP emission were collected using a 500 DCLPXR dichroic mirror with an HQ 485/30 emission filter. The EYFP emission images were collected using a 560 DCLPXR dichroic mirror with an HQ 545/40 emission filter. FRET was detected using the acceptor photobleaching approach. Two different regions within a cell expressing both proteins were selected as the region of interest (ROI), one for FRET and the other as a control. The ROI was zoomed in and bleached with the high intensity Argon 514 laser. The mean intensities of ECFP and EYFP were simultaneously recorded in the pre-bleach and post-bleach periods as a live graph. An increase in ECFP intensity following EYFP bleaching is indicative of FRET between the donor and acceptor fluorophores. The percent FRET efficiency was calculated using the formula

% FRET efficiency=1−(ECFP intensity before photobleach)(ECFP intensity after photobleach)×100
 MathType@MTEF@5@5@+=feaafiart1ev1aaatCvAUfKttLearuWrP9MDH5MBPbIqV92AaeXatLxBI9gBaebbnrfifHhDYfgasaacH8akY=wiFfYdH8Gipec8Eeeu0xXdbba9frFj0=OqFfea0dXdd9vqai=hGuQ8kuc9pgc9s8qqaq=dirpe0xb9q8qiLsFr0=vr0=vr0dc8meaabaqaciaacaGaaeqabaqabeGadaaakeaacqqGLaqjcqqGGaaicqqGgbGrcqqGsbGucqqGfbqrcqqGubavcqqGGaaicqqGLbqzcqqGMbGzcqqGMbGzcqqGPbqAcqqGJbWycqqGPbqAcqqGLbqzcqqGUbGBcqqGJbWycqqG5bqEcqGH9aqpcqaIXaqmcqGHsisldaWcaaqaaiabcIcaOiabbweafjabboeadjabbAeagjabbcfaqjabbccaGiabbMgaPjabb6gaUjabbsha0jabbwgaLjabb6gaUjabbohaZjabbMgaPjabbsha0jabbMha5jabbccaGiabbkgaIjabbwgaLjabbAgaMjabb+gaVjabbkhaYjabbwgaLjabbccaGiabbchaWjabbIgaOjabb+gaVjabbsha0jabb+gaVjabbkgaIjabbYgaSjabbwgaLjabbggaHjabbogaJjabbIgaOjabcMcaPaqaaiabcIcaOiabbweafjabboeadjabbAeagjabbcfaqjabbccaGiabbMgaPjabb6gaUjabbsha0jabbwgaLjabb6gaUjabbohaZjabbMgaPjabbsha0jabbMha5jabbccaGiabbggaHjabbAgaMjabbsha0jabbwgaLjabbkhaYjabbccaGiabbchaWjabbIgaOjabb+gaVjabbsha0jabb+gaVjabbkgaIjabbYgaSjabbwgaLjabbggaHjabbogaJjabbIgaOjabcMcaPaaacqGHxdaTcqaIXaqmcqaIWaamcqaIWaamaaa@9F3C@

## Competing interests

The author(s) declare that they have no competing interests.

## Authors' contributions

AH, SRD and SJ conceived of the study. SRD made the clones and carried out the Vpu functional assay. AH carried out the yeast two-hybrid and biochemical assays. CT carried out the confocal microscopy and FRET assays. AH and SJ wrote the paper. All authors read and approved the final manuscript.

## References

[B1] Freed EO, Martin MA, Knipe DM, Howley PM (2001). HIVs and their replication. Field's Virology.

[B2] Strebel K, Klimkait T, Martin MA (1988). A novel gene of HIV-1, vpu, and its 16-kilodalton product. Science.

[B3] Bour S, Strebel K (1996). The human immunodeficiency virus (HIV) type 2 envelope protein is a functional complement to HIV type 1Vpu that enhances particle release of heterologous retroviruses. J Virol.

[B4] Ritter GD, Yamshchikov G, Cohen SJ, Mulligan MJ (1996). Human immunodeficiency virus type 2 glycoprotein enhancement of particle budding: role of the cytoplasmic domain. J Virol.

[B5] Cohen EA, Terwilliger EF, Sodroski JG, Haseltine WA (1988). Identification of a protein encoded by the vpu gene of HIV-1. Nature.

[B6] Schubert U, Henklein P, Boldyreff B, Wingender E, Strebel K, Porstmann T (1994). The human immunodeficiency virus type 1 encoded Vpu protein is phosphorylated by casein kinase-2 (CK-2) at positions Ser52 and Ser56 within a predicted alpha-helix-turn-alpha-helix motif. J Mol Biol.

[B7] Bour S, Strebel K (2003). The HIV-1 Vpu protein: a multifunctional enhancer of viral particle release. Microbes Infection.

[B8] Bour S, Geleziunas R, Wainberg MA (1995). The human immunodeficiency virus type 1 (HIV-1) CD4 receptor and its central role in the promotion of HIV-1 infection. Microbiol Rev.

[B9] Bour S, Schubert U, Strebel K (1995). The human immunodeficiency virus type 1 Vpu protein specifically binds to the cytoplasmic domain of CD4: implications for the mechanism of degradation. J Virol.

[B10] Margottin F, Bour SP, Durand H, Selig L, Benichou S, Richard V, Thomas D, Strebel K (1998). A novel human WD protein, h-beta TrCp, that interacts with HIV-1 Vpu connects CD4 to the ER degradation pathway through an F-box motif. Mol Cell.

[B11] Bai C, Sen P, Hofmann K, Ma L, Goebl M, Harper JW, Elledge SW (1996). SKP1 connects cell cycle regulators to the ubiquitin proteolysis machinery through a novel motif, the F-box. Cell.

[B12] Fujita K, Omura S, Silver J (1997). Rapid degradation of CD4 in cells expressing human immunodeficiency virus type 1 Env and Vpu is blocked by proteasome inhibitors. J Gen Virol.

[B13] Schubert U, Anton LC, Bacik I, Cox JH, Bour S, Bennink JR, Orlowski M, Strebel K, Yewdell JW (1998). CD4 glycoprotein degradation induced by human immunodeficiency virus type 1 Vpu protein requires the function of proteasomes and the ubiquitin-conjugating pathway. J Virol.

[B14] Bour S, Perrin C, Akari H, Strebel K (2001). The human immunodeficiency virus type 1 Vpu protein inhibits NF-kappa B activation by interfering with beta TrCP-mediated degradation of Ikappa B. J Biol Chem.

[B15] Akari H, Bour S, Kao S, Adachi A, Strebel K (2001). The human immunodeficiency virus type 1 accessory protein Vpu induces apoptosis by suppressing the nuclear factor kappa B-dependent expression of antiapoptotic factors. J Exp Med.

[B16] Klimkait T, Strebel K, Hoggan MD, Martin MA, Orenstein JM (1990). The human immunodeficiency virus type 1-specific protein vpu is required for efficient virus maturation and release. J Virol.

[B17] Ewart GD, Sutherland T, Gage PW, Cox GB (1996). The Vpu protein of human immunodeficiency virus type 1 forms cation-selective ion channels. J Virol.

[B18] Callahan MA, Handley MA, Lee YH, Talbot KJ, Harper JW, Panganiban AT (1998). Functional interaction of human immunodeficiency virus type 1Vpu and Gag with a novel member of the tetratricopeptide repeat protein family. J Virol.

[B19] Paul M, Mazumder S, Raja N, Jabbar MA (1998). Mutational analysis of the human immunodeficiency virus type 1 Vpu transmembrane domain that promotes the enhanced release of virus-like particles from the plasma membrane of mammalian cells. J Virol.

[B20] Schubert U, Bour S, Ferrer-Montiel AV, Montal M, Maldarelli F, Strebel K (1996). The two biological activities of human immunodeficiency virus type 1 Vpu protein involve two separable structural domains. J Virol.

[B21] Hout DR, Gomez ML, Pacyniak E, Gomez LM, Inbody SH, Mulcahy ER, Culley N, Pinson DM, Powers MF, Wong SW, Stephens EB (2005). Scrambling of the amino acids within the transmembrane domain of Vpu results in a simian-human immunodeficiency virus (SHIVTM) that is less pathogenic for pig-tailed macaques. Virology.

[B22] Maldarelli F, Chen MY, Willey RL, Strebel K (1993). Human immunodeficiency virus type 1 Vpu protein is an oligomeric type I integral membrane protein. J Virol.

[B23] Moore PB, Zhong Q, Husslein T, Klein ML (1998). Simulation of the HIV-1 Vpu transmembrane domain as a pentameric bundle. FEBS Lett.

[B24] Xia Z, Liu Y (2001). Reliable and Global Measurement of fluorescence resonance energy transfer using fluorescence microscopes. Biophys J.

[B25] Siegel RM, Chan FK-M, Zacharias DA, Swofford R, Holmes KL, Tsien RY, Leonardo MJ (2000). Measurement of molecular interactions in living cells by fluorescence resonance energy transfer between variants of the green fluorescent protein. Sci STKE.

[B26] Schubert U, Ferrer-Montiel AV, Oblatt-Montal M, Henklein P, Strebel K, Montal M (1996). Identification of an ion channel activity of the Vpu transmembrane domain and its involvement in the regulation of virus release from HIV-1-infected cells. FEBS Lett.

[B27] Marassi FM, Ma C, Gratowski H, Strauss SK, Strebel K, Oblatt-Montal M, Montal M, Opella SJ (1999). Correlation of the structural and functional domains in the mebrane protein Vpu from HIV-1. Proc Natl Acad Sci USA.

[B28] Lopez CF, Montal M, Blasie JK, Klein ML, Moore PB (2002). Molecular dynamics investigation of membrane-bound bundles of the channel-forming transmembrane domain of viral protein U from the human immunodeficiency virus HIV-1. Biophys J.

[B29] Becker CFW, Oblatt-Montal M, Kochendoerfer GG, Montal M (2004). Chemical synthesis and single channel properties of tetrameric and pentameric TASPs (template-assembled synthetic proteins) derived from the transmembrane domain of HIV virus protein u (Vpu). J Biol Chem.

[B30] Raja NU, Jabbar MA (1996). The human immunodeficiency virus type 1 Vpu protein tethered to the CD4 extracellular domain is localized to the plasma membrane and is biologically active in the secretory pathway of mammalian cells: implications for the mechanisms of Vpu function. Virol.

[B31] Pacyniak E, Gomez ML, Gomez LM, Mulcahy ER, Jackson M, Hout DR, Wisdom BR, Stephens EB (2005). Identification of a region within the cytoplasmic domain of the subtype B Vpu protein of human immunodeficiency virus type 1 (HIV-1) that is responsible for retention in the Golgi complex and its absence in the Vpu protein from a subtype C HIV-1. AIDS Res Hum Ret.

[B32] Hengartner MO (2000). The biochemistry of apoptosis. Nature.

[B33] Rizzuto R, Duchen MR, Pozzan T (2004). Flirting in little space: the ER/mitochontria Ca^2+ ^liaison. Sci STKE.

[B34] Peterlin BM, Trono D (2003). Hide, shield and strike back: how HIV-infected cells avoid immune eradication. Nat Rev Immunol.

[B35] Kar-Roy A, Korkaya H, Oberoi R, Lal SK, Jameel S (2004). The hepatitis E virus open reading frame 3 protein activates ERK through binding and inhibition of the MAPK phosphatase. J Biol Chem.

[B36] Tyagi S, Surjit M, Kar-Roy A, Jameel S, Lal SK (2004). The ORF3 protein of hepatitis E virus interacts with liver-specific α_1_-microglobulin and its precursor, α_1_-microglobulin.bikunin precursor (AMBP) and expedites their export from the hepatocyte. J Biol Chem.

[B37] Harper JW, Adami GR, Wei N, Keyomarsi K, Elledge SJ (1993). The p21 Cdk-interacting protein Cip1 is a potent inhibitor of G1 cyclin-dependent kinases. Cell.

[B38] Bai C, Elledge SJ (1996). Gene identification using the yeast two-hybrid system. Methods Enzymol.

